# Bellevue Hospitul

**Published:** 1849-04

**Authors:** 


*Bellevue Hospitul.*—The newly finished and very convenient operating theatre, and clinical lecture room of this Hospital, was formally opened and dedicated to the united interests of science and humanity, on Friday, March 2d. 1849. The occasion called together a large number of practitioners, and a large proportion of the students of both schools, with a few intelligent medical friends of the institution. Among the latter, we observed with pleasure. several members of the Common Council, and gentlemen connected with the press. Among the medical gentlemen, besides those connected with the Hospital, we noticed Professors Smith and Watts, of the College of Physicians, Dr. Bulkley of the New York Hospital, and others.

When the audience had assembled Dr. Manly, President of the Medical Board, introduced Dr. Reese, the Resident Physician, who, at the request of his colleagues, had agreed to deliver an address on the occasion—and well did he perform this duty! After general felicitations on the happy occasion, he proceeded to point out the various and important reforms which under the supervision and with the sanction of the Honorable the Corporation, he had been able to perfect. Among these, he dwelt with earnestness and satisfaction, on the change of nurses, a change which had forever banished from the bedside of the sick and dying, the vagabonds and prostitutes from Blackwell's Island, to whom, by a most mistaken notion of economy, he had been formerly restricted in the selection of his nurses. This reform, equally important in a moral as a medical point of view, is now completed, and the Resident Physician may well felicitate the corporation and his associates, on this most auspicious change. All that was expected from it has been more than realized. He next spoke of the advantages derived from the change of locale, from the old, dilapidated buildings, to the present large and commodious hospital, the wards of which, now by recent changes, were made to compare very favorably with those of the best hospitals of our country.

He next spoke also of the facilities for clinical instruction, by a reference to the ordinance passed so far back as 1839, proved that the idea of clinical lectures had been ever present in the minds of the corporation. He showed that by various acts since passed, that this idea had never been lost sight of, and that the policy had always been to open wide the doors of this hospital to the profession, to make it subservient to the cause of science, and thus contribute to the general advance of medical education. He alluded to the various obstacles by which the realization of these truly patriotic and enlightened views had been delayed, and congratulated his hearers on the final removal of these obstacles by the reörganization of the hospital, and the appointment of a board of visiting physicians and surgeons, selected with reference to professional character only. Thus had an intelligent body of non-medical gentlemen, carried out the views recently expressed by the national medical convention, viz., that all public hospitals should be opened to professional men and students, because in the wards of hospitals only, can clinical instruction be given, and without clinical teaching, there can be no sound medical education.

And now, continued Dr. Reese, that this commodious hall has been set apart, dedicated to the advance of science and the alleviation of human suffering, we shall proceed to give a practical illustration of the benefits to be derived from our improvements, by giving you an opportunity of witnessing one of the most severe and important operations in surgery, as a pledge, that hereafter the manifold advantages of the institution should no longer be confined to the medical men officially connected with it, but, in the accomplishment of the noble design of the city government, be enjoyed by the profession at large. Dr. Reese concluded his remarks amid expressions of applause and satisfaction by all around.

Dr. Van Buren, one of the visiting surgeons, and the operating surgeon of the day, then exhibited a specimen of Encephaloid disease of the stomach, and having made some remarks on it, proceeded to state that the patient on whom the operation of lithotomy was about to be performed, was in an adjoining ward; and that before his introduction, he would be placed in a state of anesthesia by chloroform, as this possessed decided advantages over the usual mode of bringing the patient in an excited state before the audience, and then proceeding to administer this agent.

He quoted a passage from Prof. Miller's Lecture on Anesthetics to support these views. The patient, an old man, (67 years) was then brought in, perfectly insensible, and Dr. Van Buren proceeded to the operation, which was performed with skill and crowned with the most complete success. The patient was kept insensible by a continued use of the mixture of chloroform and ether, administered by Dr. Metcalf. On examining the stone, (a soft specimen of the amoniaco magnesian variety) it was found to have crystallized around a head of common grain—either rye or wheat. The presence of this foreign substance in the bladder could only be explained by supposing that it had been used to stimulate the urethra to produce onanism. Another melancholy example of human depravity and its punishment!

Dr. Mott made a few remarks on the operation while it was in progress. Dr. Reese then announced that one of the visiting physicians would next week deliver a clinical lecture in the hall, and that such instruction would hereafter be continued.

After this the assembly separated, evidently much gratified with the entire proceedings, as well as with the prospect of the future benefits to be derived from the clinical teachings, &c, of the hospital.

We cannot better close our account of this interesting day, than by the following extract from the Commercial Advertiser—it speaks, we have no doubt, the sentiments of all the more intelligent members of our community:

"We think we have cause to congratulate the public that this hospital has attained its present elevated position, under the able supervision of the medical gentlemen connected with it; for, from this great practical school of medicine and surgery will be sent forth, to fill the ranks of their noble profession, men eminently qualified to discharge faithfully and efficiently their responsible duties in whatever community they may offer themselves for the patronage of the people. And far distant be the day when political favor or censure shall do aught to dam up the stream of advantage to suffering humanity which has been thus so happily and successfully opened."

P. S.—On Friday, 10th March, the operating theatre was crowded with students and practitioners, who were much interested in a Clinical Lecture on Chronic Pleurisy, by Dr. Metcalf; after which, Dr. Van Buren amputated the arm of a man whose wrist and hand had been rendered useless, and a source of constant irritation by phlegmonous erysipelas. Dr. V. B. preceded the operation by some remarks on the necessity which compelled him him to perform it—"under no other circumstances, is an amputation justifiable. We should always remember that resorting to the knife is an acknowledgment either of our own ignorance or of the impotence of our art We cut off the limb, only because we cannot cure it.

After the operation—during which the patient was under the influence of chloroform—Dr. V. B. announced that the old man on whom he operated on the third instant, was doing well. Dr. Reese gave notice that a Clinical Lecture would be delivered on Friday, 17th, and probably an operation, lithotomy.—*Annalist.*

